# Disclosing or concealing multiple sclerosis in the workplace: two sides of the same coin—insights from a Swedish population-based survey

**DOI:** 10.3389/fpubh.2024.1331746

**Published:** 2024-02-26

**Authors:** Jessica Dervish, Victoria Mailen Arfuch, Chantelle Murley, Kyla A. McKay, Alejandra Machado, Agneta Wennman-Larsen, Emilie Friberg

**Affiliations:** ^1^Division of Insurance Medicine, Department of Clinical Neuroscience, Karolinska Institutet, Stockholm, Sweden; ^2^Psychiatry, Department of Medical Sciences, Uppsala University, Uppsala, Sweden; ^3^Division of Neuro, Department of Clinical Neuroscience, Karolinska Institutet, Stockholm, Sweden; ^4^Department of Nursing Science, Sophiahemmet University, Stockholm, Sweden

**Keywords:** multiple sclerosis, chronic disease, disclosure, concealment, work environment, survey

## Abstract

**Background:**

People with multiple sclerosis (PwMS) face health and social challenges of living with a chronic and potentially disabling condition. To disclose or conceal MS at work may critically affect individuals’ work situation, career opportunities, and health. PwMS may experience a dilemma when assessing if the possible benefits of disclosing the diagnosis outweigh the possible risks. However, concealing in the long-term may have health implications and prevent opportunities for support and work adjustments. Few studies have examined what drives PwMS to disclose or conceal MS at work and the consequences of these ways of managing MS.

**Objectives:**

To explore the reasons PwMS report for disclosing and/or concealing their MS diagnosis in the workplace, as well as the consequences they have experienced.

**Methods:**

A web-based survey of PwMS was conducted in 2021. All individuals aged 20–50 listed in the Swedish MS registry were invited to participate. The response rate was 52% and among these participants, 3,810 (86%) completed questions regarding workplace disclosure and/or concealment of MS. Free-text responses on these topics were analyzed using inductive content analysis.

**Results:**

It was common to disclose MS in the workplace (85%). Identified drivers for disclosure and concealment related to four categories: Work-related, Social, Personal and Circumstantial. Work-related drivers focused on employment or protecting one’s career, and changing one’s work situation versus maintaining it. Social drivers included the need for support, addressing or preventing stigma, and being considerate of others. Personal drivers were linked to moral values/personal beliefs and processing of the diagnosis. Circumstantial drivers related to involuntary or unforeseen events, timing factors, one’s medical condition and external opinion/advice. Identified consequences for disclosure and concealment related to three categories: Work-life, Social, and Personal. Work-life consequences included work arrangements, and career opportunities. Social consequences were linked to MS awareness, stigma, interactions and social support, as well as dynamics of work relationships. Personal consequences involved levels of disease acceptance, and attitudes toward managing MS.

**Conclusion:**

PwMS often described the question of disclosure as challenging and navigated it with caution, as both disclosure and concealment can yield favorable and unfavorable outcomes.

## 1 Introduction

Multiple sclerosis (MS) is a progressive, chronic, immune-mediated disease of the central nervous system (1). It is usually diagnosed between 20 and 40 years of age, namely during an often important stage in terms of career development. Over 20,000 persons live with MS in Sweden today, most of whom are women (2).

MS causes a wide range of visible and invisible symptoms (3), including sensory disturbances, reduced mobility, balance issues, vision loss, cognitive impairment, fatigue, pain, and depression (4). People with MS (PwMS) commonly experience bouts of neurological worsening, termed relapses (5), followed by partial or complete recovery. Nevertheless, individuals generally accumulate disability over time. These sudden as well as gradual changes in symptom severity can affect individuals’ work capacity and limit their ability to remain employed or work full-time (6–8). Hence, disclosure of the diagnosis at work is an issue of concern for PwMS. As some MS symptoms may be invisible to others (9), many PwMS can choose to conceal the disease at work (5, 10). This applies especially early in the disease when symptoms are usually milder. Accordingly, individuals may question whether the possible benefits of disclosing the diagnosis in the workplace outweigh the possible risks (9).

Disclosing MS at work can help individuals manage their symptoms and receive support from their bosses and co-workers, including appropriate work adjustments (11). Assistive equipment or software may be included in such adjustments, as could reminders or alerts if the individual requires assistance remembering tasks and meetings, flexible or reduced working hours, rest during the workday, a rearranged workspace, time off for medical appointments, parking closer to the work building, etc. (12, 13). Therefore, disclosure can be an important tool for maintaining employment and work participation (9, 14). However, disclosing a diagnosis can be a deeply personal decision, and its motivations may vary depending on the individual circumstances and preferences (9, 15). According to previous research, PwMS may disclose their diagnosis at work to explain symptoms (9), and their implications for oneself and others (15), as well as to educate others what it means to have a chronic condition (9). Disclosure has also been shown to be based on the necessity of work adjustments in order to meet job expectations, and a perceived obligation to share medical information (15). However, disclosure is commonly perceived as risky (9, 14) due to the risk of discrimination (16) and social stigma associated with chronic diseases (9, 14, 17), and can cause PwMS to conceal their diagnosis. A lack of trust in one’s employer and co-workers has also been shown to contribute to concealment, as has a lack of knowledge regarding one’s employment rights (9). Yet, studies have also shown that concealment in the long-term may cause considerable stress and affect individuals’ mental and general health and wellbeing (9, 18).

Previous studies have generally addressed either MS disclosure (9, 11, 14) or concealment (19), rarely taking both approaches into consideration. In addition, most research has focused on work-related factors associated with disclosure and concealment. Emphasis has typically been placed on a few linked aspects, such as work participation, and workplace relationships (15). Furthermore, when merely consequences of disclosing or concealing have been studied, focus has often been on clinical outcomes (20).

This research gap highlights the need for a more in-depth examination of the dynamics involved in disclosure and concealment. Specifically, there is a need for further research exploring disclosure and concealment in a more comprehensive way, taking into account that disclosure and concealment can be parallel processes. Additionally, more research is needed that examines both factors that influence the decision to disclose or conceal MS, as well as the potential outcomes, since these issues too have often been addressed separately.

Therefore, this study aims to explore the reasons PwMS report for disclosing and/or concealing their MS diagnosis in the workplace, as well as the consequences they have experienced.

## 2 Materials and methods

The present study is primarily based on free-text responses from a cross-sectional survey using a qualitative analytical approach.

### 2.1 Study population

All individuals enrolled in the Swedish MS registry between ages 20–50 were invited to answer a web-based survey concerning the work and life situation of PwMS administrated by Statistics Sweden, from May to September 2021. This study utilizes responses from one closed and five open-ended questions on disclosure and concealment in the workplace. The 66-item questionnaire was developed by the research team at the Division of Insurance Medicine at Karolinska Institutet (21). The survey also contained questions regarding sociodemographic factors and MS symptoms. Individual-level clinical data from the Swedish MS registry (SMSreg) (22) and socioeconomic data from Statistics Sweden’s Longitudinal Integrated Database for Health Insurance and Labor Market Studies (LISA) (23) database were linked. Statistics Sweden performed the data linkage and delivered anonymized data to the researchers.

Of the 8,458 adult PwMS invited, 4,412 (52%) answered the survey up to 4 reminders. Among the participants, 3,810 (86%) answered the open-ended questions about disclosure and/or concealment of their MS diagnosis in the workplace and are included in this study.

Ethical approval was received from The Swedish Ethical Review Authority (registration number: 2020–04996). Informed consent was provided by all participants by sending in the survey.

### 2.2 Survey questions

The closed survey question was: *“Have you told the following people in your workplace about your MS diagnosis?”* with the response options: (1) Yes; (2) No, but I will; (3) No; or (4) Not applicable/do not have (when the question might not fit their working circumstances, e.g., being their own boss). The no and partly no responses were collapsed for statistical purposes. We included responses that were indicated to be related to either bosses, co-workers, or both.

The five open-ended questions regarding disclosure and concealment of the participants’ MS diagnosis in the workplace were:

1. *Please share the main reason for why you have chosen to disclose* (specify to who/whom)
2. *Please share the main reason for why you have chosen not to disclose yet* (specify to who/whom)
3. *Please share the main reason for why you have chosen not to disclose* (specify to who/whom)
4. *What have been the positive or negative consequences of disclosing MS in the workplace?*
5. *What have been the positive or negative consequences of not disclosing MS in the workplace?*

It was possible for the participants to provide multiple responses regarding their boss/co-workers or both.

### 2.3 Variables

The sociodemographic variables from LISA were: sex (woman or man); type of living area (city, town, suburb, or rural area) and level of education (university or less than university). The SMSreg provided information on MS disease course (relapsing–remitting, secondary-progressive, or primary-progressive) and severity, assessed with the most recent (within 3 years of the survey date) Expanded Disability Status Scale score (EDSS). EDSS was then categorized as mild (0–2.5), moderate (3–5.5) or severe (6–9.5).

### 2.4 Data analyses

Descriptive statistics were used to summarize demographic, and clinical characteristics of the study population. The participants’ responses from the open-ended questions were analyzed using inductive content analysis (ICA) (24). The analysis was performed using Nvivo v. 11 (25). The ICA was conducted in line with the following steps (24):

1. Reading and familiarizing with the collected data. One of the researchers (JD) immersed in the data by reading the responses several times in a pre-analytical phase. First impressions and reflections were then shared with VMA, EF, and AWL, addressing the researchers’ pre-conceptions and biases. The responses varied in nature, but generally consisted of several sentences, providing detailed descriptions of the participants’ subjective experiences of disclosure and/or concealment. In this coding process, responses from bosses and co-workers were handled separately, then codes were compared to determine whether there were any differences in content. The responses pertaining to bosses and co-workers were, however, generally similar, why we have not separated them in the results below.
2. Identifying and generating initial codes. Given the large sample, coding was performed as a sequential and iterative process. JD did the initial coding and analysis with the support of VMA. After having fully coded and analyzed 400 individuals’ responses, EF and AWL separately coded 50 different individuals each out of these 400 individuals in order to contrast the findings, test reliability, and guarantee methodological rigor. Thereafter, JD fully coded an additional 600 individuals (1,000 in total). The remaining 2,810 participants’ responses were then read but only coded when new meaningful conceptual units emerged, or richer quotes were identified to support existing codes. During this process, preliminary codes were revised, redefined, removed or merged considering the incoming data in the analysis.
3. Developing categories and subcategories. JD and VMA inductively organized and grouped the identified codes into overarching conceptual units (categories and subcategories). The results were condensed into six tables, where the different categories, subcategories, and representative quotes were comprehensively presented.
4. Refining categories and subcategories. After that, EF and AWL reviewed the suggested categories and subcategories and after discussions with JD and VMA the results were presented to all authors. Discrepancies in relation to coding and categorizations were solved through consensus discussion between all co-authors.
5. Defining and naming of categories. After discussion among all co-authors, categories and subcategories were named. The drivers and consequences of disclosing or concealing were then tabulated, respectively.
6. Producing the study. The results were summarized by JD with the support of VMA and the supervision of EF, AWL, CM, AM, and KAM. Representative quotes were translated from Swedish to English for the purposes of publication by CM and checked by all co-authors.

## 3 Results

Of the 3,810 individuals who were included in the study, the majority were women (*n* = 2,724; 71.5%). Most (84.9%) reported having disclosed their diagnosis to their boss and/or co-workers. Of these, 71.5% had disclosed their MS to both their boss and co-workers, 9.6% had only disclosed to their co-workers and 3.9% had only disclosed to their boss. The majority of participants were aged 40–49 (51.3%) and had low disability (EDSS score: 0–2.5). Demographic data of participants are displayed in Table 1.

**Table 1:** Demographic data of the participants.

	Total (*N* = 3,810)
Age group (years)	
20–29	303 (8.0%)
30–39	1,239 (32.5%)
40–49	1953 (51.3%)
50–51	315 (8.3%)
Sex	
Women	2,724 (71.5%)
Men	1,086 (28.5%)
University level education	
Yes	2,441 (64.1%)
No	1,369 (35.9%)
Type of MS	
Relapsing–remitting	3,570 (93.7%)
Primary progressive	62 (1.6%)
Secondary progressive	146 (3.8%)
Missing	32 (0.8%)
MS severity	
Low (EDSS = 0–2.5)	2,935 (77.0%)
Moderate (EDSS = 3–5.5)	443 (11.6%)
Severe (EDSS = 6–9.5)	88 (2.3%)
Missing	344 (9.0%)
Disclosed	
Yes	3,238 (84.9%)
No	572 (15.1%)

Generally, the responses for bosses and co-workers were similar, however, it was noticeable that responses for bosses tended to be more formal and work-focused at times, while responses for co-workers tended to be more social. In the analysis, the participants’ reported reasons for disclosure and concealment were interpreted as reflecting underlying drivers. We conceptualized a driver as the force that initiates, maintains, supports, and stops individuals’ behavior in regard to disclosure and concealment; whether internal or external.

The results are presented in two sections: first the identified drivers for disclosing and concealing MS, second the identified consequences of disclosing and concealing MS.

### 3.1 Drivers for MS disclosure and concealment

The main categories of disclosure and concealment were found to be related to work-related, social, personal, and circumstantial drivers (Table 2).

**Table 2:** Overview of main categories with related generic categories: drivers for disclosure and concealment in the workplace.

Disclosure	Main categories	Concealment
Generic categories		Generic categories
To seek work adjustments and occupational safety	**Work-related drivers** Enabling continued work participation, ensuring financial security, and protecting career opportunities	To protect daily work and employment status
To protect career and prestige		To protect career and prestige
To justify/explain work-related absence		To protect future economic stability
To manage the disease at work		
To prevent being stigmatized/socially judged	**Social drivers** Seeking social support and understanding, preventing stigma, and being considerate of others	To prevent being stigmatized/socially judged
To have or to seek social support		To avoid an unsupportive work environment
To prevent the social impact of MS		To prevent the social impact of MS
Being guided by values and emotions	**Personal drivers** Being guided by values, processing MS, preserving or reorienting identity, and maintaining personal wellbeing	MS & me: *“My MS is a private matter”*
Moving toward MS acceptance		Ongoing emotional processing of MS
Seizing an opportunity to disclose	**Circumstantial drivers** Considering timing aspects, medical/work conditions, external influence, and involuntary/unexpected events	Not the right time to disclose
Not deemed necessary to conceal MS at work		Not deemed necessary to disclose MS at work
Due to MS symptoms		
Disclosure driven by external event/someone else		Concealing in relation to others’ advice
Disclosing in relation to others’ advice		

#### 3.1.1 Work-related drivers: enabling continued work participation, ensuring financial security, and protecting career opportunities

Work-related drivers were highly prevalent among the responses. Despite appearing to be two opposite ways of handling MS, participants who disclosed and concealed sought—in different ways—to pursue employment or to protect their work-life. Participants who disclosed their diagnosis often sought to *change* their current situation or conditions at work, whereas participants who concealed the diagnosis sought to *maintain* them.

By disclosing MS, participants recognized the possibility of adjusting their work situation to better manage their health, including a modified workload, more flexible working hours, adequate working conditions, and a secure workplace. Furthermore, participants expressed that they disclosed so that others in the workplace would know what to do if they became ill during working hours. MS was also disclosed to enable rest during the workday and avoid the pressure of being social with co-workers. In addition, participants disclosed to request sickness benefits and justify absences from work for medical appointments.

*“Because I have cognitive impairment, I cannot cope with open office environments and therefore had to adapt my workplace by sitting in a smaller room with fewer disturbances.”* (Woman, 41 years, EDSS = 0, disclosed).

By sharing the diagnosis, participants described that they hoped that others would intervene if they were about to push their own limits. Additionally, disclosure made it easier for participants to decline responsibilities and promotions at work—ensuring they did not take on more than they could handle, both from their own and their boss’ perspective.

*“The combination of wanting too much, loving your job, having a diagnosis and at the same time a personality that is ‘prone to burnout’ is not recommended. I need people around me who can understand, but also make sure to stop me in time.”* (Woman, 42 years, EDSS = 2, disclosed).

Concealment, in turn, was found as a strategy to prevent negative work-related outcomes. In these cases, participants sought to retain their employment, avoid career obstacles, or protect their professional reputation by sidestepping MS-related discrimination and stigma.

*“My assessment is that it would be an aggravating circumstance when looking for new positions or tasks. That they would rather bet on someone they do not expect will soon deteriorate physically and cognitively.”* (Man, 45 years, EDSS = 1, concealed).

Both disclosure and concealment were described as stemming from a need to secure career prospects, but in different ways. Participants stated that by disclosing, they could make meaningful use of the MS experience in their daily work, especially if working within the health field. MS was also disclosed when participants needed their employer to verify whether they could pursue a particular profession. However, from a concealment perspective, it was thought that securing one’s career aspects was best achieved by keeping the diagnosis a secret. In many cases, this was due to an assumption that disclosing MS at work could have negative short- and long-term consequences.

*“Of course, I’m also afraid of losing my job and finding it difficult to get a new job at another workplace if it gets out that I have the diagnosis.”* (Woman, 47 years, EDSS = 1.5, concealed).

Feeling insecure within the labor market, in the short or long-term, also contributed to concealment, e.g., by having a precarious or temporary employment, being newly hired in the workplace, and not wanting to alarm one’s boss.

#### 3.1.2 Social drivers: seeking social support and understanding, preventing stigma, and being considerate of others

Disclosing or concealing the MS diagnosis in the workplace was often described as a socially anchored decision; one that put focus on support, status, and social impact. The participants expressed that they cared or were concerned about their relationships at work and how they were portrayed socially by others. Sometimes the decision to disclose or conceal MS was driven by caring more about the perceived or assumed social needs of others than their own.

Seeking social support and understanding emerged as an influential driver for disclosing MS—either by actively seeking it or as a result of working in a caring and trusting environment. Having a supportive work environment was referred to as a “safe space” that facilitated disclosure. Other motivations included gaining others’ understanding of MS-symptoms and their impact on work ability, as well as receiving informal assistance with daily tasks if necessary.

*“So that they would understand why I sometimes shake a lot, find it difficult to walk, lose my speech and have a poor eyesight.”* (Woman, 29 years, EDSS = 2, disclosed).

Whether to disclose or conceal the diagnosis was often reported as a dilemma, as it could lead to both favorable and unfavorable outcomes. Participants were not necessarily confronted with this dilemma only at work, but in their wider lives too. They expressed that part of it was due to the hardening societal climate, excessive work demands, and prevalent inequalities that strike those who are already in a vulnerable position in the labor market disproportionately hard.

*“Overall, I feel like we have a climate in society right now where you are ‘damned if you do, damned if you do not’. You’re expected to be tough and talk about your troubles so many people can relate to you – but on the other hand, you will be seen as an attention seeker and whiny by others if you do.”* (Woman, 29 years, EDSS = 2, disclosed).

Furthermore, the fear of being stigmatized was a common driver for concealment. The perceived risks associated with disclosing MS at work involved being treated differently, socially excluded, pitied, judged, gossiped about or discriminated against. Participants who concealed also mentioned being afraid that MS could damage their professional image.

*“I’m a high-achiever and want others to see me as such. Whenever I tell others about my disease, they react with surprise and that’s what I want. Since I was diagnosed, I’ve done everything not to be associated with it.”* (Woman, 42 years, EDSS = 1.5, concealed).

Participants sometimes claimed they had nothing to gain by disclosing their diagnosis, which was usually related to having an unsupportive work environment. There was a resoluteness that the risks of revealing MS would outweigh any potential benefits. Past experiences of being targeted for discrimination or insensitive remarks about the MS diagnosis at work upon disclosure often contributed to this positioning.

*“Unfortunately, I’ve had bad experiences of being discriminated against and fired because of my MS […]. This has made me now view it as my right to keep my disease private. It simply hurts too much to be rejected/discriminated against because of something that I did not choose myself to bring into my life.”* (Woman, 44 years, EDSS = 1.5, concealed).

Moreover, disclosure was attributed to protect the business or the company one worked for, to give one’s boss an opportunity to make an informed decision regarding the employment, or to prevent unnecessary worry from co-workers when being absent or experiencing a relapse. Similarly, concealment was adopted to avoid worrying other people, being considered a burden at work, or making others uncomfortable. The latter stance was usually motivated by having mild symptoms and receiving an effective treatment.

#### 3.1.3 Personal drivers: being guided by values, processing MS, preserving or reorienting identity, and maintaining personal wellbeing

Disclosing or concealing MS in the workplace was often described as the result of an inner process, where moral values or personal beliefs helped guide one’s actions. The importance of being honest was usually expressed with the common saying *“honesty is the best policy.”* Based on the participants’ answers, disclosure was made from a sense of commitment to truth-telling, where transparency was the right course of action regardless of its consequences. Accordingly, being open about the diagnosis was seen as a way of living authentically according to one’s core values.

*“I believe it’s important not to hide my diagnosis, mostly because my daughter has type 1 diabetes and we have always worked for her not be ashamed of her disease and so I cannot live differently. In my job we work with work environment issues and discrimination, so it’s important for me to stand up for who I am.”* (Man, 42 years, EDSS = 1, disclosed).

In some cases, participants reported no conflict in disclosing MS at work, with a seemingly neutral positioning toward it and some even referred to it in almost trivial terms. It was evident from these responses that MS was already an accepted part of the participants’ lives, and that neither the disease itself, nor the symptoms or their impact on their work ability, were factors to be ashamed of.

*“There’s no reason not to say anything? It’s as natural as talking about the weather.”* (Woman, 37 years, EDSS = 0, disclosed).

Sometimes, disclosing the diagnosis was described as having a therapeutic effect that facilitated MS acceptance. *“MS is a part of me”* and similar expressions, were often used indicating an internal process of not only accepting but also embracing the diagnosis. However, some noted this as happening in parallel with a sense of loss and sadness that they carried over their former selves. The need to emotionally process MS was expressed as a driver for both disclosure and concealment. While some participants claimed that they had begun to accept the diagnosis as a permanent part of their lives, others struggled to initiate this process.

*“I am not and will never again be the same person. I used to work around 200% and drove at that pace in everything (…). So, going from all that to now barely being able to work 75% has been a life crisis to say the least.”* (Woman, 36, EDSS = 2.5, disclosed).

In some cases, participants conveyed being to some extent in denial and holding on to their previous identity without MS. The process of adjusting to being chronically ill was often difficult and they described dealing with internal resistance. Participants who concealed commonly drew a clear line between having a diagnosis and *being* a diagnosis, arguing that MS did not define them as a person but was merely a condition they were unfortunate to endure. Concealment was sometimes referred to by the participants as a strategy to protect their interests and maintain a sense of normality in life by keeping their usual routines, activities, and relationships going. Moreover, it was mentioned how concealment diverted attention from the disease and served as means of focusing on other aspects of one’s life unrelated to MS. Some participants reported that they used concealment as a way to convince themselves they could still live their former lives and manage their disease. By visualizing themselves as healthy and acting accordingly, they rose above their circumstances and the adversities of living with MS.

*“I just do not want to; I want to be the same person regardless.”* (Woman, 23, EDSS = 1, concealed).

On the contrary, some participants with a mild disease progression disclosed to change preconceptions about the disease and reported that they found meaning in contributing to social change, inside as well as outside of the MS community. The identified drivers included wanting to increase MS knowledge, normalize the disease, break MS-related social stigma, become a role model for people with chronic conditions, and encourage others on health-improving lifestyle changes. Participants expressed that since they experienced that MS was often associated with greater impairments and disabilities than they had personally experienced, they wanted, or saw it as their responsibility, to challenge such notions. Although participants who concealed also expressed the wider social benefits of talking openly about MS, they were not yet ready themselves to do so. Other times, participants described finding a middle way, where it was still possible for them to increase knowledge and challenge preconceptions of MS without necessarily disclosing. This was done by pretending to talk about a friend or a relative’s experiences of living with MS, instead of their own. In this way they could avoid potential negative reactions at work.

*“I’m very healthy in my MS and happy to be an ambassador to shed light on the disease. There’s a lot of public ignorance about it and if I can show how healthy and strong one can be with MS, that’s great.”* (Woman, 36 years, EDSS = 0, disclosed).

Furthermore, concealment was adopted when participants preferred handling MS as a private matter. Here, participants mentioned not wanting to entrust others with such intimate information, especially since they were not legally obligated to do so. In this sense, concealment was often led by a feeling of discomfort discussing MS, or to be reminded of it, and as a need to protect one’s mental health.

#### 3.1.4 Circumstantial drivers: considering timing aspects, medical/work conditions, external influence, and involuntary/unexpected events

In some cases, disclosing the diagnosis was not described in terms of an active choice. Instead, the participants claimed that circumstantial factors guided, or even determined, whether to disclose or conceal the diagnosis. Additionally, it was evident that participants were influenced by other people’s opinions and advice, and that they sometimes acted in a reactive rather than proactive manner when it came to disclose or conceal MS.

In disclosure scenarios, participants reported that they seized a convenient opportunity to share their health condition in discussions with others that concerned the same or similar subject. There were also participants who claimed neither hiding nor openly displaying the diagnosis. Here, the situation itself determined the course of action. Often in these scenarios, disclosure happened by chance, rather than by intention.

*“I do not see much reason to keep it to myself, although I also do not make an effort to let everyone that I come into contact with know about it.”* (Man, 37 years, EDSS = 1, disclosed).

In other scenarios, disclosure occurred beyond participants’ control, when unforeseen circumstances, or another person urged the diagnosis to be revealed. Sometimes, the MS progression and its unpredicted symptoms did not offer room for planning when and how to address it. Occasionally, disclosure was undesirably driven by someone else, without the individuals’ consent.

*“It was informed to my two immediate managers and the HR department in connection with my sick leave for exhaustion, since my doctor wrote it in my medical certificate against my will.”* (Woman, 42 years, EDSS = 2, disclosed).

The Covid-19 pandemic was another unexpected cause for disclosing the diagnosis at work. Several reasons were involved: to ensure social distancing in the workplace, being allowed to work from home, as well as motivating their vaccination status.

Moreover, timing and work relations were other important factors. Not considering the present moment the right time to disclose, being unfamiliar with or not close enough to people at work were frequent drivers for concealment. At times, participants concealed because they claimed others had not yet asked them any health-related questions. Further, some participants had concealed their diagnosis for so long, they felt that it was too late to disclose it.

Advice from others played a role in whether participants disclosed or concealed MS and was commonly received by a health professional, their boss, or another influential contact. In some cases, participants were advised to be cautious since disclosure could potentially negatively affect their employment and career prospects. In other cases, participants were encouraged to disclose the diagnosis, mainly due to the disease’s slow progression and effective treatments.

*“I was advised by my doctor not to tell because there are many misconceptions about MS that could affect my professional life, such as career opportunities [...].”* (Man, 45 years, EDSS = 0, concealed).

The disease itself was also described as an important circumstance. Some reported that they did not believe disclosure was necessary due to mild MS symptoms or an unaffected work ability, and that it had the potential of doing more harm than good. However, participants were often open to share their diagnosis if, and when, circumstances changed.

### 3.2 Consequences for MS disclosure and concealment

The main categories of consequences of disclosure and concealment were found to be work-related, social, and personal factors (Table 3).

**Table 3:** Overview of main categories with related generic categories: consequences of disclosure and concealment in the workplace.

Main categories	Positive consequences generic categories		Negative consequences generic categories	
	Disclosure	Concealment	Disclosure	Concealment
**Work-life consequences:** Work adjustments, working situation, and career aspects	Opened up new career opportunities	Protected work participation and professionalism	Felt/been undermined professionally at work	Been viewed as unproductive
	Received work adjustments		Been subjected to workplace mistreatment	Missed out on work adjustments
**Social consequences:** Dissemination of MS knowledge, MS-related stigma, workplace treatment, social support, and emotional reactions of others	Improved social interactions	Prevented being stigmatized and discriminated because of MS	Experienced the stigma associated with MS	
	Gained social support and understanding		Lacked social support and understanding	Experienced negative impact on social relationships
	Experienced positive social impact of disclosure		Experienced excessive/unwanted involvement by others regarding MS	Have not raised MS awareness
			Encountered misguided acts of kindness at work	
**Personal consequences:** Disease acceptance, personal development, maintaining the façade, emotional stressors, and integrity issues	Developed self-empowerment	Maintained a sense of normality in life	Experienced work pressure	Experienced work pressure
	Experienced emotional ease upon disclosure	Been able to use concealment as a disease management strategy	Disclosure has raised difficult emotions	Concealment has raised difficult emotions
	Contributed to one’s own acceptance of MS	Been able to protect privacy		Felt lonely/isolated at work
	Preserved sense of identity before MS	Preserved sense of identity before MS		Been burdened by the MS secret at work

#### 3.2.1 Work-life consequences: work adjustments, working conditions, and career aspects

The consequences that participants reported as a result of disclosure and concealment were often centered on their work-life situation, usually focusing on work adjustments, working conditions, and career opportunities.

In terms of positive work-related consequences, a clear difference was observed for disclosure and concealment: while participants who disclosed generally expressed appreciation for the fact that their work situation had *changed*, participants who concealed expressed that they valued the ability to *maintain* their work situation and prevented professional losses.

A positive consequence of disclosure involved receiving work adjustments. For instance, participants reported benefiting from reduced or flexible working hours, as well as individualized tasks and responsibilities. Being transparent about MS also made it easier to be absent from work when attending medical appointments. Work adjustments related to the Covid-19 pandemic were further brought up, including the opportunity to work remotely, and having work schedules adjusted in a way to minimize physical contact at work. In addition, participants occasionally found new or more suited career opportunities following disclosure, either through promotions or assistance in finding a new position. Being granted a permanent position upon disclosing MS was also mentioned. Positive consequences of protecting work-life and career development were further noted when concealing MS. Participants stated that since their work efforts were not overshadowed by the diagnosis, there was not a risk of others having doubts about or questioning their work ability. Concealment was then perceived to make it possible to continue pursuing career goals.

*“That my boss understands my disease and how it affects me. It has made it possible for me to control my work more based on the daily schedule and arrange the work so that it suits me better. As long as I meet my deadlines, I’m completely in control.”* (Man, 40 years, EDSS = 2, disclosed).

*“I continue to present myself as a professional employee. Not a patient/burden/weak. I’m not missing out on opportunities to advance in my career, as my achievements speak for themselves.”* (Woman, 30 years, EDSS = 0, concealed).

A reported negative consequence of disclosure was being exposed to pessimistic assumptions about the disease which lowered others’ expectations of one’s work performance. Participants claimed being treated as ‘less capable’. A loss of autonomy at work was described and an inability to make decisions regarding work capacity. Other negative consequences involved being deprived of work tasks and responsibilities, which hindered professional development. A further example was not receiving job offers owing to gossip or misconceptions about one’s health.

*“I’ve realized in retrospect that many people associate my disease with reduced work capacity linked to limited thinking activity and/or other work performance. You simply feel that your career opportunities have disappeared because everyone still thinks that you’ll ultimately end up in a wheelchair/be bedridden and therefore not be capable of long-term work.”* (Man, 43 years, EDSS = 4, disclosed).

Some participants also expressed that their work-related needs were neglected after disclosing the diagnosis and that they did not receive expected adjustments nor the assistance they had expected from their bosses, which in turn had a negative impact on their work and health. Participants further stated that they were negatively surprised that they had not experienced a decrease in workload or demands upon disclosure; and sometimes, even the opposite was described. Another negative and unexpected consequence of disclosing was to not being allowed to work from home.

For participants who concealed, on the other hand, they knew they could not count on any type of work adjustments when having MS symptoms that negatively impacted their work performance. Concealment made them unable to explain why they could not focus on the same type of tasks as before, or why they sometimes needed rest. Consequently, they sometimes had to work under unhealthy conditions, including being assigned too many tasks and responsibilities and having difficulty balancing work and personal life.

*“They see me as a high achiever and impose far too many tasks and responsibilities, which means that all my free time is spent recuperating.”* (Woman, 41 years, EDSS = 0, concealed).

Similar to what was reported by those who disclosed the diagnosis, a negative consequence of concealment was also related to being exposed to health-threatening work conditions during the pandemic. Concealment further led to difficulties in scheduling medical appointments and treatments during working hours. Participants also described discrimination at work following disclosure, most of which were attributed to their bosses. Being laid off from work shortly after disclosing MS was mentioned. Some also had contractual pressure to maintain a certain level of work performance, otherwise they would be fired. Some claimed being subjected to offensive behavior and violations of their work rights upon disclosing MS, such as repeated insults and degrading remarks. Others further described being urged to resign by their bosses, despite having an unaffected work ability. At times, participants were questioned by their bosses for not disclosing MS at an earlier time or accused of deceiving their bosses due to this reason. Moreover, it was stated that the diagnosis was sometimes used by bosses as an excuse to cover up, or not deal with, workplace deficiencies.

*“My boss has actively worked to get me to resign through* var*ious forms of offensive treatment. I’ve been punished by not receiving a salary increase, been accused of things I did not do even though I was able to prove that my boss was wrong. I have not received the aids I needed at work. I’ve been told time and time again that I’m worth nothing as a person and as an employee, and that they do not want me employed.”* (Woman, 47 years, EDSS = 6, disclosed).

#### 3.2.2 Social consequences: dissemination of MS knowledge, MS-related stigma, workplace treatment, social support, and emotional reactions of others

The reported consequences of disclosing and concealing the diagnosis at work were often linked to social aspects, such as interactions with others in the workplace, the presence or absence of social support, as well as work relationship characteristics.

A positive consequence of disclosing the MS-diagnosis was having the ability to increase knowledge about the disease and contribute to address as well as potentially reduce its stigmatizing status. Participants described that by talking openly about MS, they could show others the great variance in how people are affected by the disease, and the different ways in which they can effectively cope with MS. An additional positive consequence reported was that disclosure often came as a surprise, or even a shock, to people at work, which in itself challenged a common notion that MS is a noticeable and highly disabling condition.

*“The disease does not get as stigmatizing. They see that I function like anyone else despite the disease.” (Man, 39 years, EDSS = 1, disclosed).*

Often, concealment led to positive outcomes such as retaining social inclusion at work and avoiding stigma and discrimination that can be associated with the disease. By concealing, participants further reported that they could maintain appearances and keep their social status intact, which also protected them from becoming subjected to gossip or pity. However, for many, the greatest benefit was still being seen for who they were before MS.

For participants who disclosed, receiving social understanding was regarded as a positive consequence, as their health concerns were validated and taken into consideration by people at work. As a result of talking openly about MS, they were able to have a dialog about their work limitations with their bosses and coworkers. Furthermore, it was viewed as positive having people at work showing interest and curiosity regarding the diagnosis. Receiving kind and uplifting comments upon disclosure was something that participants reported that they did not expect and were pleasantly surprised by.

Moreover, participants described an improved sense of cohesion, openness in communication and an improved social bond after disclosing MS. It appeared that by being vulnerable about personal struggles, others felt comfortable to do the same. Participants also expressed that by talking openly about their MS journey, they had inspired others to healthier lifestyles. When they could contribute to the wellbeing of others, participants expressed that they found meaning in living with MS and could make sense of the hardships they had experienced. Comparatively, participants who concealed their diagnosis reported that taking this approach limited their opportunities for social understanding. It was often described how concealment led to the loss of, and inability to build, personal relationships at work.

*“Some co-workers have opened up about their own situation and in this way, we have become closer to each other.”* (Man, 34 years, EDSS = 2.5, disclosed).

*“Difficult with closer contacts with co-workers. Sometimes difficult to get an understanding of my experiences with the disease. Details of my life history are missing.”* (Woman, 35 years, EDSS = 2, concealed).

As for negative consequences of disclosure, participants reported that they did not feel understood or empathized by their boss or co-workers. Situations of social exclusion at work were also reported, as were subjection to jokes, bullying and other forms of humiliation. It was also reported that the diagnosis was often downplayed and treated insensitively as a result of others’ misbelief and skepticism. In addition, different forms of excessive or unwelcome involvement by others were commonly experienced as the diagnosis tended to evoke strong reactions. Participants for example claimed being compared to other people’s MS experiences in an insensitive way, where people at work made negative generalizations regarding the diagnosis. Another consequence was being perceived and treated by others as sicker than they were, and that they felt increasingly confined to their disease. Participants further reported having experienced misplaced acts of kindness at work upon disclosing MS, such as receiving excessive help at work or others’ overly considerate behavior in moments where it was not needed.

Receiving unsolicited MS advice was also mentioned, which was considered as intrusive since it undermined their own ability to manage their disease. Furthermore, participants who disclosed claimed that their privacy was invaded at work and that people did not respect their boundaries, such as sharing their medical information with others. The opposite was often stated by those who concealed the diagnosis, where a positive consequence was precisely having the ability to maintain such personal integrity. In this regard, concealment provided a sense of autonomy and control over whether, when, to whom, and how they disclosed their diagnosis.

*“People who aren’t familiar with the disease often think that it manifests itself in the same way for everyone. By knowing someone who has it, they assume that it’ll be the same for me. But the disease is highly individual, with different degrees, which people often have no idea about whatsoever. This bothers me.”* (Woman, 33 years, EDSS = 0, disclosed).

#### 3.2.3 Personal consequences: disease acceptance, personal development, maintaining the façade, emotional stressors, and integrity issues

Disclosure and concealment were often described as resulting in profoundly personal consequences, that touched on degree of disease acceptance, as well as personal attitudes and approaches to MS. The participants reported both personal wins and losses of disclosing or concealing the diagnosis, which was also related to how they handled MS; some were more emotionally based, while others were more of a principal matter.

Upon disclosure, participants sometimes reported a greater confidence, acceptance, and trust in themselves, as well as an increased self-understanding. Seizing the opportunity to share personal development through the MS journey and highlighting positive aspects of living with the diagnosis were further emphasized. Other positive consequences entailed being praised at work for the courage of disclosing MS, experiencing a greater ownership in terms of health and learning how to set firm(er) boundaries toward others at work. Among the positive consequences of MS concealment, on the other hand, the ability to maintain a sense of normality was frequently reported; offering participants distraction from the hardships of living with MS. When comparing this reasoning from the one provided for disclosure, a telling similarity was the strive for taking command of the MS situation and to turn it to one’s advantage—through means of self-empowerment in the former, or the ability to regulate emotions in the latter.

*“In a certain way I’ve become more confident, and I do not have to feel the same shame, stress and inadequacy as I did when I was working full-time and trying to hide my diagnosis.”* (Woman, 40 years, EDSS = 2, disclosed).

*“That situations with co-workers are more natural and as they were before I was diagnosed. I do not need to feel ‘sick’ or that there’s something wrong with me when discussing work, hobbies,* etc. *In general, life feels more normal when not too many people are informed about my diagnosis and the health-related focus that follows.”* (Man, 29 years, missing EDSS, concealed).

As for negative consequences of concealment, it required participants to keep up a façade of good health at work and choose their words carefully to not reveal the diagnosis. The constant self-monitoring required to maintain concealment was referred to as an energy drain. Having no one to share their experiences with or who could offer social support when needed was also described. In retrospect, the participants realized they could have received more understanding, social safety, and not have had to become so self-reliant, if they had disclosed. Many expressed that they longed for a support system they did not have.

*“Hiding my feelings, having worries, living up to a façade that absolutely cannot be broken. This is how I’ve lived for many years.”* (Man, 41 years, EDSS = 0, concealed).

Accordingly, a positive consequence of disclosing was not having to keep MS a secret and a relief to be able to talk openly about it. Participants reported feeling better emotionally as they were not required to maintain a healthy façade at work. By allowing themselves to let their guard down, they entered a more self-accepting phase in their MS journey. The opposite was described by those who concealed, where participants expressed feelings of guilt and shame for hiding their MS. The inability to advocate for the disease was also stated as a negative consequence, where participants felt that they were insincere; violating the sense of being true to both themselves and others for not disclosing MS.

*“A relief came once I had told, after many hours of consideration and thinking. In a way, it feels easier to move on and a part of accepting that I have this diagnosis.”* (Woman, 30 years, EDSS = 0, disclosed).

A positive consequence of concealment often raised was that concealing in itself functioned as a way of managing the disease. Concealing helped create a mindset where obstacles were viewed as challenges rather than as threats. Participants reported that due to a consistent strategy of not paying more attention than necessary to the diagnosis, they felt less sick. In these scenarios, participants appeared resolute that a mind-over-matter perspective was in their favor, regardless of the personal costs involved.

In some cases, the consequences mentioned by the participants were more linked to the emotions that disclosure or concealment gave rise to. A fear of personal repercussions, such as becoming a target for stigma, stereotyping, prejudice, or discrimination sometimes arose after disclosure. Such fears could have existed long before disclosure, but were intensified afterwards. As it was described, disclosing MS was analogous to a sense of defeat. A described fear among participants who disclosed was also that they would be met with misbelief from others given their symptoms often were invisible. A fear of misbelief was also expressed by those who concealed but for being misinterpreted as lazy or unproductive when, in fact, having MS symptoms. Not being able to correct these misconceptions was reported as a source of stress.

*“It was very hard emotionally for me to mention it. I still do not know to this day why it is/was so hard and distressing. Part of me did not want them to know, part of me did not want them to see me differently and I think part of me was almost ashamed of being sick.”* (Woman, 35 years, EDSS = 2.5, disclosed).

Furthermore, those who disclosed shared, as a negative consequence, that they were worried that they would be considered a burden in the workplace; not seen as good enough employees or not contributing sufficiently to the organization. Participants reported that due to their disclosure, they tended to put pressure on themselves to perform well at work. They also described not expressing workload limitations, despite the detrimental effects on their health. Similarly, those who concealed expressed that because of this decision, they tended to strive to perform at the level of healthy individuals, even when struggling with severe MS symptoms, despite knowing such ambitions were unrealistic and could come at a high personal cost.

*“I have to work and carry out tasks as if I was a healthy person and sometimes I have to fight and keep it together even though I feel like my body could collapse.”* (Woman, 32 years, EDSS = 3, concealed).

## 4 Discussion

In this survey-based study of 3,810 participants, we identified drivers and consequences of disclosure and concealment of MS in the workplace.

Notably, we discovered that although disclosing or concealing initially seemed to be two very different approaches to manage MS in the workplace, the identified drivers in both types of scenarios were often similar; representing two sides of the same coin. Both those who disclosed and those who concealed expressed similar needs: to feel included, to be seen for the person they were, and to be someone to count on, irrespective of their diagnosis.

Comparing the drivers for disclosure and concealment, we often observed strategic characteristics. Participants who disclosed MS often sought to improve their work situation, whether it be work adjustments, a more flexible schedule, an adapted workload, or improved social bonding with their co-workers. In contrast, participants who concealed MS often sought to maintain their work situation as much as possible. By adopting this approach, they could safeguard their career development, maintain their sense of self, and be in control of how they were perceived by others.

Similar to a previous study (26), participants who stated having mild symptoms sometimes concealed the diagnosis. As can be expected, concealment was found more challenging or impossible among those individuals who stated having more severe symptoms or higher disability, given the substantial impact of MS in their everyday life. Lack of social support and fear of stigma, discrimination, and employment termination were also common drivers for concealment. Concealment was typically seen as preferred over disclosure when participants feared being socially excluded at work due to their illness.

Stigma was a recurring factor for both disclosure and concealment—in the former case by actively seeking to increase the knowledge about MS and reduce its stigmatizing status, and in the latter case by actively avoiding being potentially subjected to it.

Weighing disclosure or concealment was typically a carefully thought-out process; often influenced by the individuals’ past experiences. Participants reported feeling more secure disclosing their diagnosis when certain conditions existed, such as having a responsive boss, understanding co-workers, or possibilities for an adjusted work environment. In workplaces where the opposite was described, there was often hesitancy to disclose. Participants were usually reluctant to re-disclose if they had not received support and understanding at work in the past. As previously reported (9), it was clear that the work atmosphere could either be a facilitator for disclosure or a barrier against it, which signals the importance of contextual factors in disclosure as well as concealment decisions.

Moreover, we observed that, in some cases, the consequences of disclosing or concealing MS in the workplace aligned with the drivers. For example, participants described disclosing the diagnosis to ease their work situation, which was also the generated outcome. Similarly, participants described concealing the diagnosis to continue being seen for the person they were before they were diagnosed with MS, as indeed they were.

There were participants who recalled disclosure as an overall positive experience, which had led to a clearer understanding of needs or greater support in the workplace. Conversely, there were others who recalled disclosure as an overall negative experience, which had led to diminished or even ignored work needs, social exclusion, discrimination, or difficulties in advancing in the career. Similar differences were reported for concealment experiences. Most frequently, however, consequences of disclosure and concealment were not described as strictly positive or negative, but usually included elements of both. Mixed experiences were expected and have been reported previously (15). Participants who disclosed the diagnosis could, for example, receive work adjustment but at the same time experience reduced trust in their work ability or suffer social exclusion from others in the workplace. Likewise, participants who concealed the diagnosis could continue building their careers but also experience feelings of not being honest with others. Regardless of having disclosed or concealed, participants usually expressed some form of compromise or sacrifice.

In accordance with previous research (27, 28), we observed that participants expressed a need and appreciation for social support in the workplace both prior to and following disclosure. However, overly considerate behavior from bosses and co-workers had a discouraging, belittling effect, and could turn into interference. This emphasizes the difficulty for others to navigate what kind of support PwMS need at different stages of the disease course.

Since MS is both a chronic and fluctuating disease, disclosure and concealment are often reoccurring events in the lives of PwMS (18)—even if they have disclosed previously, they may face new and different symptoms or new situations. Consequently, decisions regarding disclosure and concealment need to be revisited as both the disease and working conditions can change over time. Disclosure and concealment decisions can also be expected to shift character along the way; what may be irrelevant to share about one’s medical condition at one time may be directly decisive at another. Therefore, disclosure and concealment should be understood as a continuing process. To disclose or conceal the MS diagnosis at work can be a difficult decision and may affect individuals’ work situation, career opportunities, disease management and, ultimately, their health and disease progression (18). Our findings demonstrate the complexity inherent in both disclosing and concealing MS, and their respective consequences.

To address disclosure and concealment together rather than separately was beneficial, especially since these can be parallel processes for PwMS in the workplace. This allowed us to gain a more nuanced understanding of their effects on PwMS and uncover the complex interplay between disclosure and concealment. The results further stress that disclosure and concealment both encompass advantages and disadvantages.

### 4.1 Methodological considerations and future research

To the best of our knowledge, no prior study has investigated both drivers and consequences for disclosure and concealment in a workplace context, qualitatively with a dataset of this magnitude. A strength of this study is the large number of participants who responded to the open-ended questions in the survey, which have also enabled a broad and representative spectrum of working-aged PwMS. Using such a large amount of survey data has allowed a variety of perspectives and experiences to be captured on this study topic. The response rate was reasonably high despite the very comprehensive questionnaire, which indicated that the participants considered these topics to be of importance and convenient to answer. Furthermore, the use of Nvivo (25, 29) software program in the analyses enabled detailed coding and good transparency. A further strength is the multi-professional and inter-disciplinary research group analyzing the data from different perspectives, including medicine, epidemiology, psychology, public health, health economics, nursing, and social anthropology.

The study also had limitations. First, while we were able to capture a range of experiences through the open-ended question format, using a survey, we did not have the opportunity to clarify or follow-up the participants’ answers. Future research could explore PwMS’ lived experiences on these matters through in-depth interviews.

Of the total participants (52%) who responded to our survey, a majority (85%) reported having disclosed their MS diagnosis in the workplace. It is important to acknowledge that we do not know whether the ones that responded to the survey were more or less likely to disclose, the prevalence of disclosure in the workplace may thus be different in the overall MS population from what our results suggests. However, in our qualitative analyses we had sufficient responses from individuals who had concealed their diagnoses, and our sample represented a diverse range of individuals with various backgrounds and experiences.

It should be further noted that most of our participants had low levels of physical disability. Nonetheless they had still disclosed their diagnosis at work. Therefore, the results may be more transferrable to individuals with mild MS, or those with “invisible symptoms” and not reflect experiences of those with more severe forms of the disease which are more observable. As it may not be possible to conceal more severe symptoms, disclosing or concealing the diagnosis may not always be an active choice. Our results also contrast with those of other studies in this research area, which suggest that PwMS often conceal the diagnosis when having invisible symptoms (5), or a mild form of the disease (10).

Further factors impact generalizability. The employee protections provided by the Swedish “Employment Protection Act” (30) may to some part explain the large number of PwMS who disclosed their diagnosis in our study, even when having mild or no disability. This relatively high job security in comparison to many other jurisdictions, may make PwMS more comfortable disclosing their diagnosis at work. Moreover, this study was conducted during the Covid-19 pandemic. This may have influenced the perceived need among PwMS to disclose their diagnosis at work.

It would also have been interesting to consider the impact of occupational and clinical variables. These variables, such as type of work, occupational sector, company size, and time since diagnosis, could potentially influence individuals’ decision to disclose or conceal their MS in the workplace. Future research should take these factors into account to gain a more comprehensive understanding of workplace disclosure and concealment.

Furthermore, as has been pointed out before, disclosure and concealment can be seen as ongoing processes. Considering the cross-sectional nature of our study, we were unable to fully determine how matters relating to disclosure and concealment unfold over time. Studies with a longitudinal design are thus needed to further clarify the relationship between drivers and consequences.

## 5 Conclusion

Among our 3,810 participants, a majority had disclosed MS in the workplace. However, PwMS often described it as challenging and navigated it with caution. This is because disclosure and concealment are often complex and sensitive issues for which there are rarely any straight answers with both potentially yielding favorable as well as unfavorable outcomes. Consequently, it is important to recognize the dilemma frequently faced by PwMS in this regard, and avoid the assumption that disclosure is always the ideal course of action. Although a supportive work environment could help facilitate open dialog, it does not necessarily mean that PwMS will feel comfortable disclosing their diagnosis in this particular setting. Therefore, employers as well as organizations as a whole, should not only focus on creating a supportive work environment for their employees, but should also actively work toward promoting a culture of open communication. This could be achieved in various ways, for example by demonstrating inclusive behavior, fostering an acceptive work atmosphere by having zero tolerance of judgmental attitudes, engaging in employee well-being and health, providing the option of anonymous reporting, and offer support and resources to those who do wish to disclose their diagnosis. Ultimately, to create a truly inclusive and supportive work environment, it is vital to respect each individual’s decision whether or not to disclose their diagnosis.

## Data availability statement

The datasets presented in this article are not readily available because the data used in the study cannot be made public. Such data can only be made available, after legal review, to researchers who meet the criteria to access such sensitive and confidential data, according to the General Data Protection Regulation, the Swedish Data Protection Act, the Swedish Ethical Review Act, and the Swedish Public Access to Information and Secrecy Act. Readers may contact Associate Professor EF (emilie.friberg@ki.se) regarding this data.

## Ethics statement

The studies involving humans were approved by the Swedish Ethical Review Authority (registration number: 2020–04996). The studies were conducted in accordance with the local legislation and institutional requirements. The participants provided informed consent to participate in this study.

## Author contributions

JD: Conceptualization, Formal analysis, Writing – original draft, Writing – review & editing. VA: Conceptualization, Formal analysis, Writing – review & editing. CM: Conceptualization, Writing – review & editing. KM: Conceptualization, Supervision, Writing – review & editing. AM: Conceptualization, Supervision, Writing – review & editing. AW-L: Conceptualization, Methodology, Supervision, Writing – review & editing. EF: Conceptualization, Funding acquisition, Investigation, Project administration, Resources, Supervision, Visualization, Writing – review & editing.
